# Dexamethasone in osteogenic medium strongly induces adipocyte differentiation of mouse bone marrow stromal cells and increases osteoblast differentiation

**DOI:** 10.1186/s12860-015-0056-6

**Published:** 2015-03-13

**Authors:** Olfa Ghali, Odile Broux, Guillaume Falgayrac, Nathalie Haren, Johannes PTM van Leeuwen, Guillaume Penel, Pierre Hardouin, Christophe Chauveau

**Affiliations:** Lille2-ULCO, PMOI, F-62200 Boulogne-sur-Mer, France; Department of Internal Medicine, Erasmus MC, 3000 CA Rotterdam, The Netherlands; PMOI, ULCO, Boulevard Napoléon, BP 120, 62327 Boulogne-sur-mer, Cedex France

**Keywords:** Bone marrow stromal cells, Cell differentiation, Dexamethasone, Co-differentiation medium, Osteoblast, Adipocyte, Mouse

## Abstract

**Background:**

Osteoblasts and adipocytes share a common mesenchymal stem cell origin. Therefore, it has been suggested that the accumulation of marrow adipocytes observed in bone loss is caused by a shift in the commitment of mesenchymal stem cells from the osteogenic pathway to the adipogenic pathway. Supporting this hypothesis the competition between adipogenic and osteogenic lineages was widely demonstrated on partially homogeneous cell populations. However, some data from mouse models showed the existence of an independent relationship between bone mineral content and bone marrow adiposity. Therefore, the combination of adipogenesis and osteogenesis in primary culture would be helpful to determine if this competition would be observed on a whole bone marrow stromal cell population in a culture medium allowing both lineages.

In this aim, mouse bone marrow stromal cells were cultured in a standard osteogenic medium added with different concentrations of Dexamethasone, known to be an important regulator of mesenchymal progenitor cell differentiation.

**Results:**

Gene expression of osteoblast and adipocyte markers, biochemical and physical analyses demonstrated the presence of both cell types when Dexamethasone was used at 100 nM. Overall, our data showed that in this co-differentiation medium both differentiation lineages were enhanced compared to classical adipogenic or osteogenic culture medium. This suggests that in this model, adipocyte phenotype does not seem to increase at the expense of the osteoblast lineage.

**Conclusion:**

This model appears to be a promising tool to study osteoblast and adipocyte differentiation capabilities and the interactions between these two processes.

## Background

In bone diseases such as osteoporosis, it has been observed an increase in the content of bone marrow adipocytes at the expense of bone volume [1-3]. Two main explanations were proposed. On the one hand, adipocytes could express antiosteoblastic factors [4,5]. On the other hand, because the both cell types share a common mesenchymal stem cell (MSC) progenitor [6-8], the involvement of a competition between these two differentiation pathways in the decrease of osteoblastic cell population described in osteoporotic bone specimen is often evoked. Nevertheless, some data, like independent alterations of bone mineral content and bone marrow adiposity in some mouse models [9], led to discuss more these hypotheses. Indeed, several key questions remain to be answered. First, antiosteoblastic factors from adipocytes remain to be identified. Second, the mechanism responsible for cell orientation to adipogenic or osteogenic lineage in the bone marrow is still poorly defined [9,10]. Third, the competitive relationship between these two pathways in vivo is still discussed particularly because of the existence of independent pre-osteoblastic and pre-adipocytic cell populations in the bone marrow [11].

Therefore, the combination of adipogenesis and osteogenesis in primary culture would be helpful not only to identify modulators of the osteoblast/adipocyte balance or to evaluate cell differentiation capacities, but also to determine if the antagonism between adipocytes and osteoblasts, often described on a selected cell population, would be observed in a more heterogeneous population of bone marrow stromal cells (BMSCs).

Among molecules used in culture protocols to induce cell differentiation, Dexamethasone (Dex), a synthetic glucocorticoid, is known to be an important regulator of mesenchymal progenitor cell commitment to osteoblast, adipocyte and chondrocyte lineages. Indeed, it has been demonstrated, in many studies, that Dex regulates the osteogenesis of human MSCs and mineralization *in vitro* [12-17]. It is also demonstrated that Dex has a pro-osteogenic effect on mouse MSCs [18].

The involvement of Dex in the regulation of human adipogenesis was described in many works [19,20]. It has been shown that in human MSCs, continuous Dex treatment augmented adipocyte differentiation in adipogenic medium, and osteoblast differentiation in osteogenic medium [21]. Dex is also used for adipocyte differentiation of mouse BMSCs [22]. In addition, Grigoriadis et al. [23] showed on culture of clonal cell populations from rat calvaria that Dex added to osteogenic medium induced adipocytic differentiation while allowing osteoblastic phenotype. Thus, we wondered whether Dex might be used to allow both osteogenesis and adipogenesis in murine BMSCs. Therefore, in the present study, we investigated the effect of different concentrations of Dex added to osteogenic medium on adipocytic and osteoblastic differentiation of mouse BMSCs. We showed that in this medium only Dex at 100 nM induced adipocyte phenotype and increased osteoblast phenotype.

## Results

### Addition of Dex at 100 nM in osteogenic medium strongly induced adipogenesis

**Figure 1:**
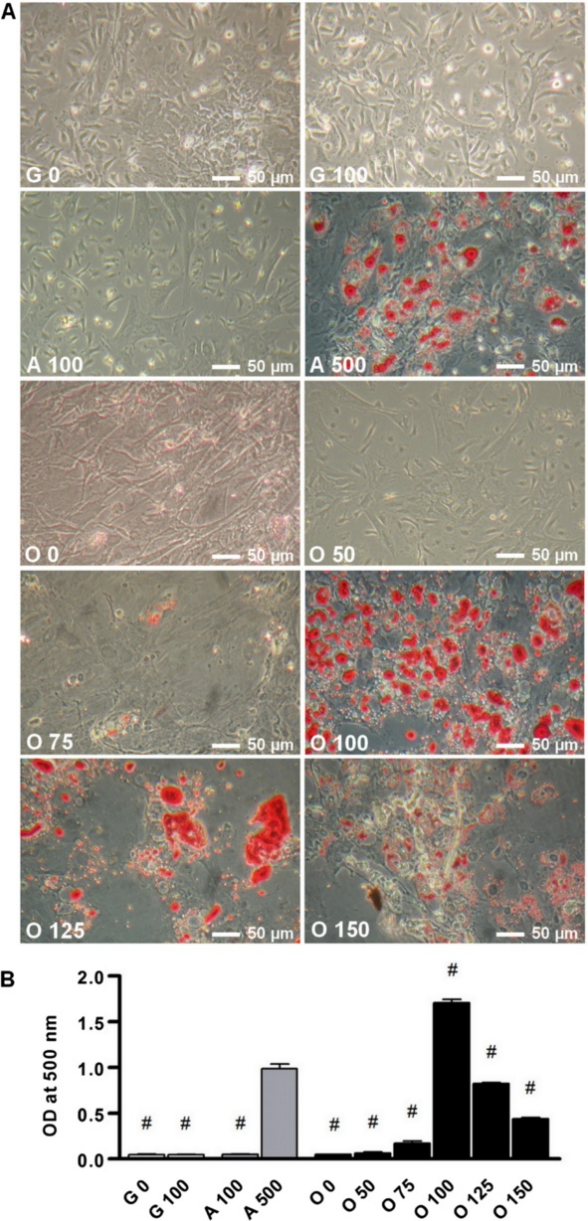
**Dex addition in standard osteogenic medium induces adipocyte differentiation.** Confluent BMSCs were cultured for 14 days in growth medium (G 0), in growth medium added with Dex 100 nM (G 100), in adipogenic medium containing Dex 100 nM (A 100) or 500 nM (A 500), or cultured in osteogenic medium supplemented with 0 to 150 nM of Dex (O 0 to O 150). **(A)** The effect of Dex on adipocyte differentiation was determined by oil red O staining. Representative images from four independent experiments are presented here. **(B)** Relative quantification of lipid droplet formation was measured at 500 nm after oil red O staining and compared to adipogenic medium results. Representative data from four independent experiments are shown as means +/− S.E.M. ^#^ *p* < 0.05 vs. A 500 medium.

To investigate the effect of Dex on differentiation, BMSCs were cultured for 14 days in osteogenic medium supplemented or not with different amounts of Dex ranging from 50 nM to 150 nM. Oil red O staining revealed the gradually appearance of lipid droplets in media supplemented with 75 and 100 nM (Figure 1A) while higher doses in osteogenic medium (125 and 150 nM) seemed to decrease the total number of cells during the culture according to microscopic observations. Optical density of the extracted oil red O revealed that the presence of Dex at 100 nM in osteogenic medium induced a higher lipid accumulation than 100 nM and even 500 nM of Dex in the adipogenic medium itself (Figure 1B).

**Figure 2:**
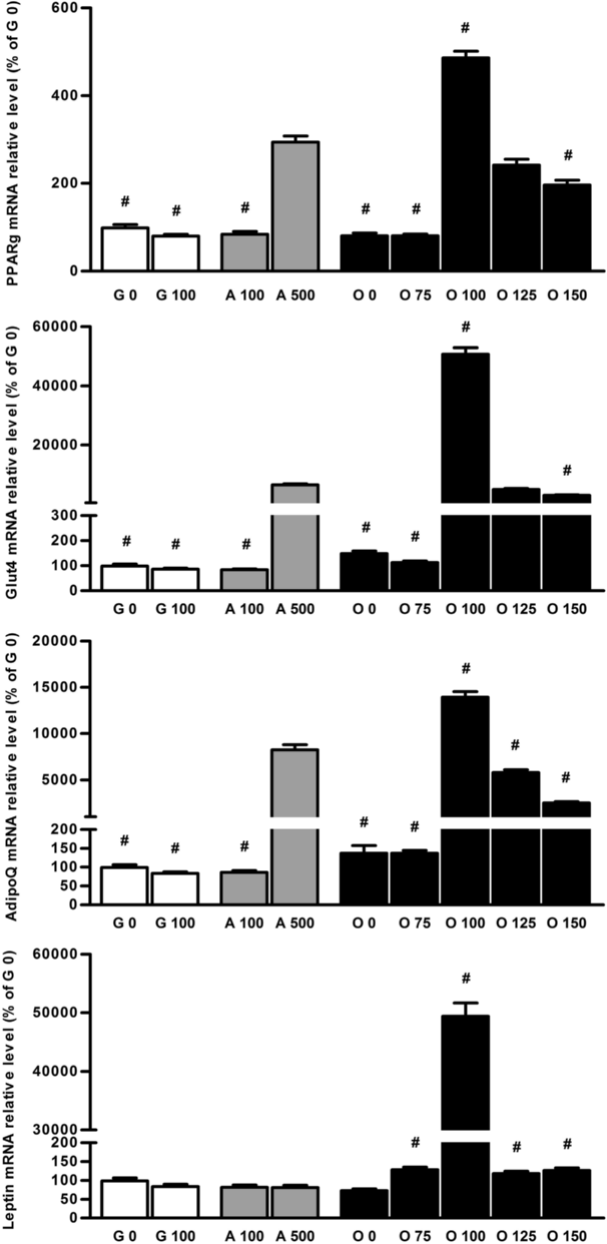
**Dex addition in standard osteogenic medium induces adipocyte marker expression.** Relative levels of mRNA adipocyte markers (PPARgamma, glut4 (glucose transporter type 4), adiponectin (AdipoQ) and leptin) were determined on BMSCs cultured for 14 days in growth medium (G 0), in growth medium added with Dex 100 nM (G 100), in adipogenic medium containing Dex 100 nM (A 100) or 500 nM (A 500), or cultured in osteogenic medium (O 0) or osteogenic medium supplemented with 75 to 150 nM of Dex (O 75 to O 150). Levels of adipocyte marker mRNAs were reported to the mean of mRNA level of two housekeeping genes Gapdh2, and 18S and expressed in percent of cells cultured in growth medium. Representative data from four independent experiments depicted as means +/− S.E.M. are shown. ^#^ *p* < 0.05 vs. A 500 medium.

Enhancement of adipocyte differentiation in this medium was confirmed by the induction of mRNA of early and late differentiation markers PPARgamma, glut4 (glucose transporter type 4), AdipoQ (adiponectin) and leptin. As shown in Figure 2, Dex added at 100 nM to the osteogenic medium induced the highest adipocyte differentiation because it enhanced the mRNA expression of all these markers at the highest level. Surprisingly, leptin expression was strongly increased only by Dex 100 nM in osteogenic medium when compared with all other media. Even standard adipogenic medium with Dex 500 nM did not induce an increase in leptin expression. This was not due to the high concentration of Dex because also in a modified adipogenic medium with only 100 nM of Dex, no increase in leptin expression was observed (Figure 2). This suggests a more complicated regulation of leptin expression in this model.

**Figure 3:**
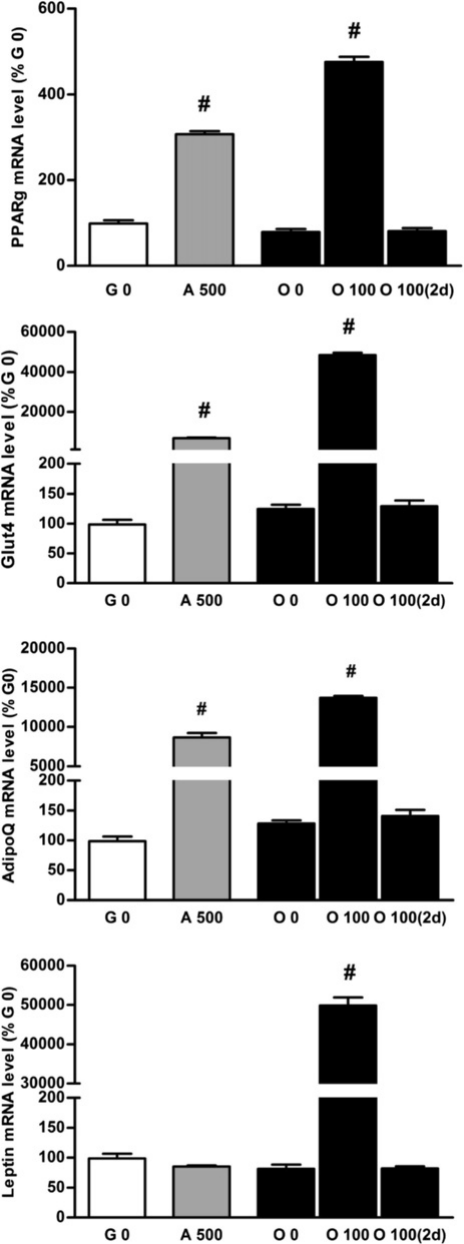
**Dex addition in standard osteogenic medium for the last 2 days has no effect on adipocyte differentiation.** Relative levels of mRNA adipocyte markers (PPARgamma, glut4 (glucose transporter type 4), adiponectin (AdipoQ) and leptin) were determined on BMSCs cultured for 14 days in growth medium (G 0), in adipogenic medium containing Dex 500 nM (A 500), or cultured in osteogenic medium (O 0) or osteogenic medium supplemented with 100 nM of Dex (O 100) for 14 days or for the last 2 days (O 100(2d)). Levels of adipocyte marker mRNAs were reported to the mean of mRNA level of two housekeeping genes Gapdh2, and 18S and expressed in percent of cells cultured in growth medium. Representative data from four independent experiments depicted as means +/− S.E.M. are shown. ^#^ *p* < 0.05 vs. O 0 medium.

To verify that adipocyte marker expression in osteogenic medium supplemented with Dex 100 nM was not only due to enhancement of some gene promoters by Dex, cells were cultured in osteogenic medium for 14 days and 100 nM of Dex were added only for the last 2 days. This addition of Dex did not modify the mRNA expression of adipocyte markers when compared to standard osteogenic medium (Figure 3), confirming the full commitment of cells to the adipocyte differentiation pathway in O 100 medium.

### Addition of Dex at 100 nM in osteogenic medium also potentiated osteogenesis

**Figure 4:**
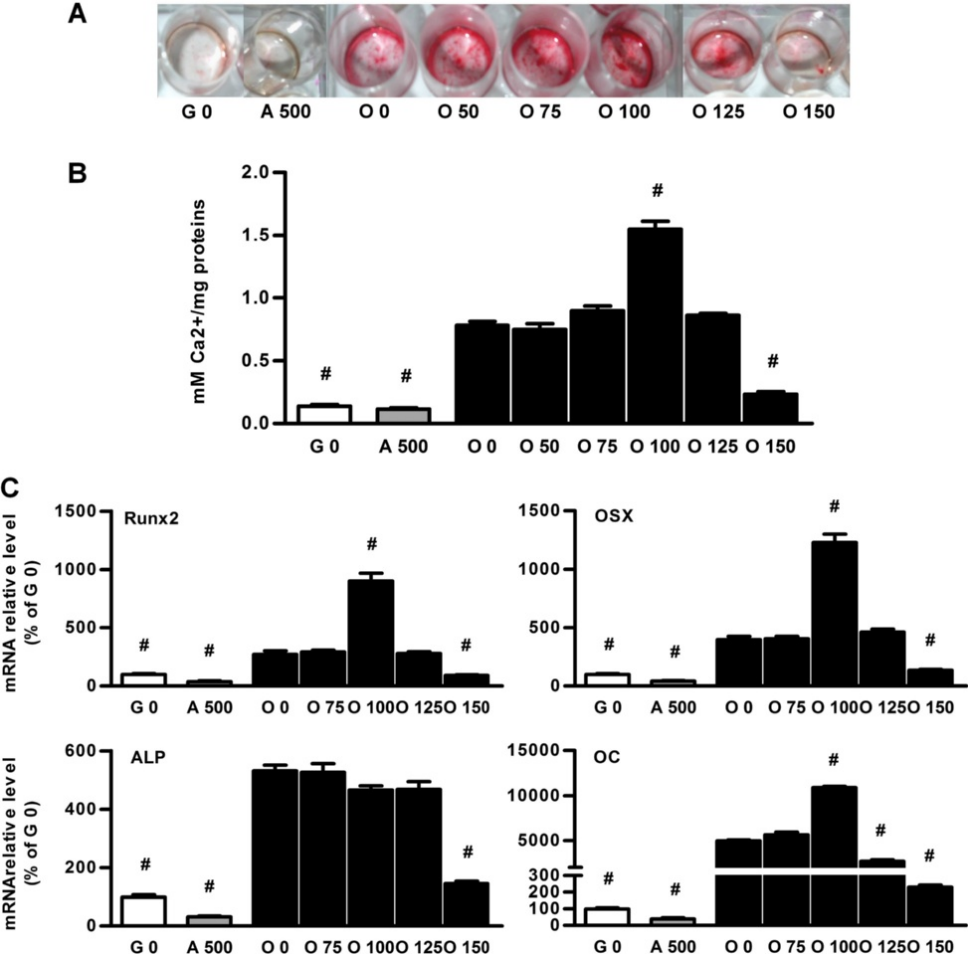
**Dex addition in standard osteogenic medium potentiates osteoblast differentiation.** Osteoblast differentiation was tested on BMSCs cultured for 14 days in growth medium (G 0), in adipogenic medium containing Dex 500 nM (A 500), or cultured in osteogenic medium (O 0) or osteogenic medium supplemented with 75 to 150 nM of Dex (O 75 to O 150). **(A)** Mineralized nodules were highlighted by alizarin red staining. Representative images from four independent experiments are presented here. **(B)** Mineralization was quantified by measuring the calcium to protein ratio and compared to osteogenic medium results. Representative data from four independent experiments are shown as means +/− S.E.M. **(C)** Relative levels of mRNA osteoblast markers (Runx2, OSX (osterix), ALP (alkaline phosphatase), OC (osteocalcin)) were determined. Results were reported to expression of the mean of Gapdh2 and 18S and expressed in percent of cells cultured in growth medium. Representative data from four independent experiments depicted as means +/− S.E.M. are shown. ^#^ *p* < 0.05 vs. O 0 medium.

The impact of Dex on osteoblast differentiation was also determined. Alizarin red staining showed that BMSCs mineralized in osteogenic medium when Dex was added at concentrations ranging from 0 to 125 nM (Figure 4A). Interestingly, our results demonstrated that the calcium to protein ratio was higher in osteogenic medium added with Dex 100 nM than in usual osteogenic medium (Figure 4B).

Furthermore, quantitative RT-PCR results demonstrated that when compared to usual osteogenic medium, only 100 nM of Dex in osteogenic medium induced an up-regulation of osteoblast marker mRNA expressions (runx2, osteocalcin (OC) and osterix (OSX)) but without any effect on the expression of the alkaline phosphatase (ALP) (Figure 4C). We concluded that Dex added at 100 nM induced the strongest osteoblast differentiation. Thus, we selected this concentration for the further experiments performed in co-differentiation medium.

**Figure 5:**
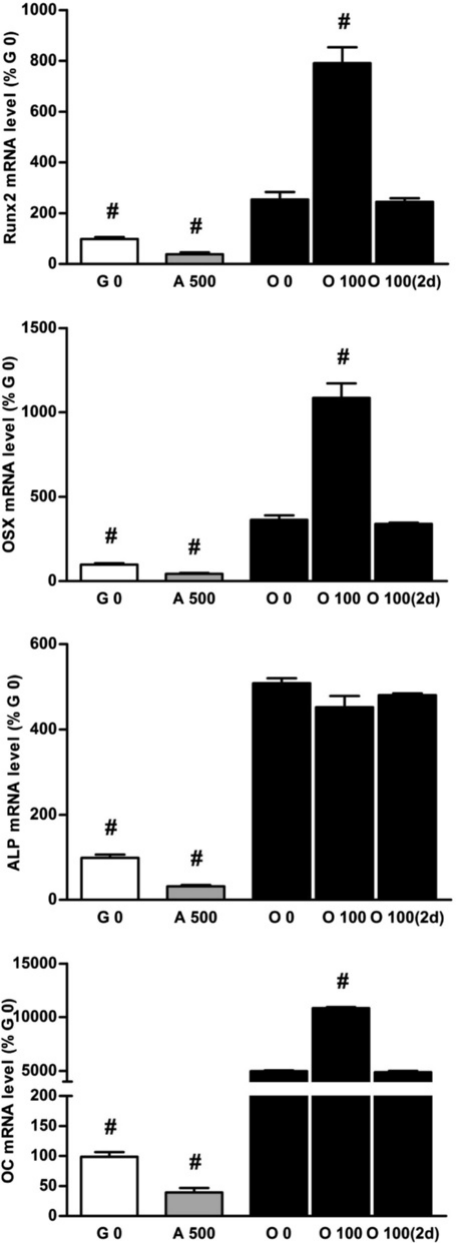
**Dex addition in standard osteogenic medium for the last 2 days has no effect on osteoblast differentiation.** Relative levels of mRNA osteoblast markers (Runx2, OSX (osterix), ALP (alkaline phosphatase), OC (osteocalcin)) were determined on BMSCs cultured for 14 days in growth medium (G 0), in adipogenic medium containing Dex 500 nM (A 500), or cultured in osteogenic medium (O 0) or osteogenic medium supplemented with 100 nM of Dex (O 100) for 14 days or for the last 2 days (O 100(2d)). Results were reported to expression of the mean of Gapdh2 and 18S and expressed in percent of cells cultured in growth medium. Representative data from four independent experiments depicted as means +/− S.E.M. are shown. ^#^ *p* < 0.05 vs. O 0 medium.

Likewise the mRNA expression of adipocyte markers, addition of Dex 100 nM for the last 2 days of the 2 week culture in osteogenic medium did not modify the mRNA expression of osteoblast markers (Figure 5). This could indicate that the enhancement of osteoblast markers in co-differentiation medium when compared to osteogenic medium was not only due to a direct effect of Dex on some gene promoters.

**Figure 6:**
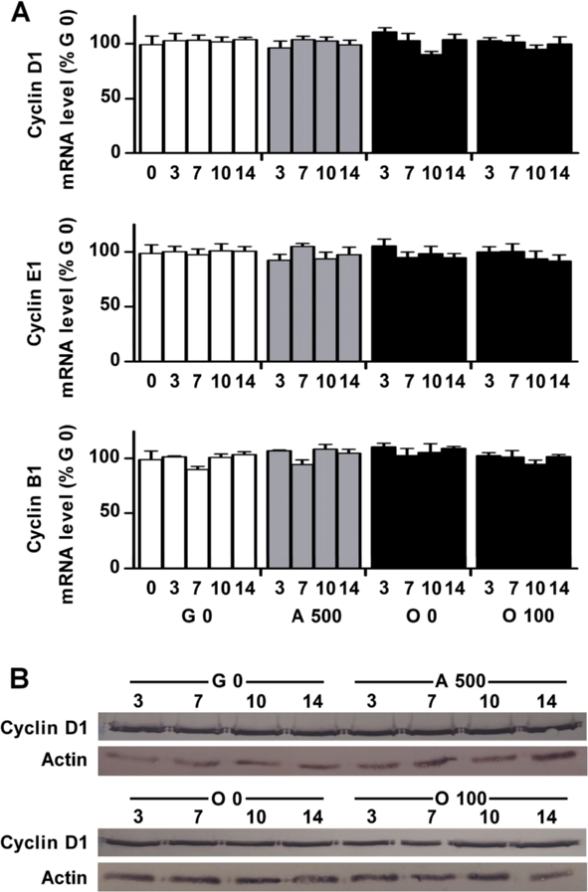
**Dex addition in standard osteogenic medium did not affect cell proliferation. (A)** Dex effect on cell growth was determined by measuring the mRNA expression of 3 cyclins (D1, E1 and B1) on BMSCs cultured for up to 14 days in growth medium (G 0), in adipogenic medium containing Dex 500 nM (A 500), or cultured in osteogenic medium (O 0) or osteogenic medium supplemented with 100 nM of Dex (O 100). Results were reported to expression of the mean of Gapdh2 and 18S and expressed in percent of cells cultured in growth medium. **(B)** Dex effect on cell growth was assessed by performing western blot analysis of cyclin D1 in the same conditions as in **(A)**.

To determine if the increased levels of differentiation markers were due to Dex effects on cell proliferation, the relative quantification of mRNA cyclins (D1, E1 and B1) was performed by quantitative RT-PCR after 3/7/10/14 days of culture in growth medium (G0), adipogenic medium with 500 nM of Dex (A 500), osteogenic medium (O 0) and co-differentiation medium (O 100). Dex addition did not alter the mRNA level of these cyclins (Figure 6A). This suggested that Dex does not have any effect on cell proliferation in our model. To strengthen this hypothesis, we performed western blot analysis of Cyclin D1 (Figure 6B). These further experiments showed no obvious differences at the protein level. Altogether, these data led us to suppose that in our model, Dex acts by inducing higher differentiation levels in the two lineages.

### Characterization of mineralization nodules and lipid accumulation by Raman microspectroscopy in the co-differentiation medium

**Figure 7:**
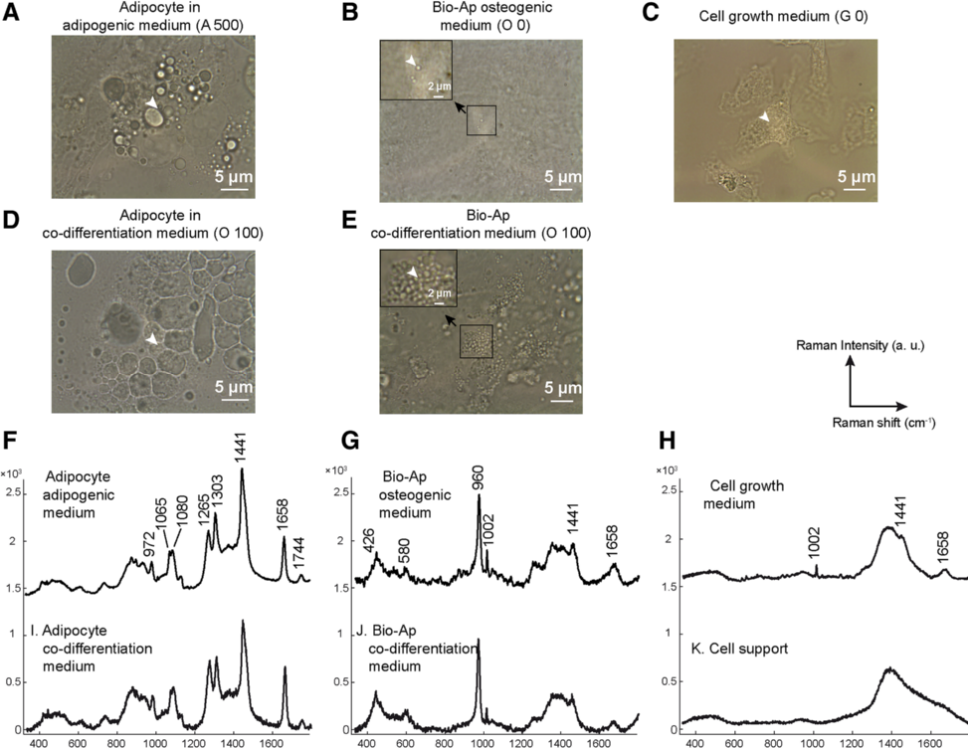
**Characterization of mineralization nodules and lipid accumulation by Raman microspectroscopy in the co-differentiation medium.** BMSCs cultured for 14 days in osteogenic, adipogenic (with Dex 500 nM) or co-differentiation media (100 nM of Dex added to osteogenic medium) were analyzed by Raman microspectroscopy. Optical images representative of three independent experiments performed in triplicate are shown. **(A)** Lipid accumulation of adipocytes in the adipogenic medium. **(B)** Mineralized nodules of apatitic calcium phosphate named here bioapatite (Bio-Ap) of cells cultured in the osteogenic medium. **(C)** Undifferentiated cells in the growth medium. (**D**) Lipid accumulation of adipocytes of cells cultured in the co-differentiation medium. **(E)** Mineralized nodules of Bio-Ap of cells cultured in the co-differentiation medium. **(F)** Reference Raman spectra of lipid accumulation of adipocytes in the adipogenic medium; **(G)** Reference Raman spectra of mineralized nodules of Bio-Ap in the osteogenic medium; **(H)** Reference Raman spectra of undifferentiated cell in the growth medium; **(I)** Raman spectra of lipid accumulation of adipocytes in the co-differentiation medium; **(J)** Raman spectra of mineralized nodules of Bio-Ap of cells cultured in the co-differentiation medium; **(K)** Reference Raman spectra of cell support. The position of the Raman spectrum is indicated by white arrow heads; a.u. = arbitrary units.

To characterize the nodules observed in the co-differentiation medium and to confirm lipid accumulation in functional adipocytes, we used the Raman microspectroscopy. In this aim, we determined reference Raman spectra of cells differentiated in adipogenic and osteogenic media respectively. Indeed, the adipocyte phenotype of cells cultured in adipogenic medium for 14 days was observed by optical image which demonstrated presence of lipid droplets (Figure 7A). The reference Raman spectra of these cells with lipid droplets showed the presence of characteristic bands of adipocytes as those described in the study of Downes et al. [24]. These bands were found at 972, 1065, 1080, 1265, 1303, 1441, 1658 and 1744 cm^−1^ (Figure 7F). They were not detected in undifferentiated cells (cells grown in growth medium) and cell support (dish without cells) (Figures 7C, H and K).

Osteoblast phenotype of cells cultured in osteogenic medium for 14 days was determined by presence of nodules (Figure 7B). Raman study of these nodules showed spectra characteristic of apatitic calcium phosphate as those found in previous studies [24,25], with bands observed at 426, 580 and 960 cm^−1^ (Figure 7G). They were not detected in undifferentiated cells and cell support. The assignment of all bands is specified in Table 1.

**Table 1:** **Raman bands observed in the osteogenic, adipogenic and co-differentiation media**

**Osteogenic medium**	**Adipogenic medium**	**Co-differentiation medium**	**Assignments**
426		426	ν_2_ PO_4_
580		580	ν_4_ PO_4_
960		960	ν_1_ PO_4_
	972	972	δ (=C-H) out-of-plane
1002		1002	Phenylalanine/HPO_4_
	1065	1065	ν(C-C)
	1080	1080	ν(C-C)
	1265	1265	δ (=C-H) lipids
	1303	1303	δ(CH_2_) twisting lipids
1441	1441	1441	δ(CH_2_) scissor lipids/proteins
1658	1658	1658	ν(C = C) lipids/proteins
	1744	1744	ν(C = O) in CH_2_-COOR

Assignments are based on studies from Downes et al. [24].

Optical images of cells cultured in co-differentiation medium for 14 days showed the presence of lipid droplets (Figure 7D) and nodules (Figure 7E) as those observed in adipogenic and osteogenic media respectively. Interestingly, the Raman analysis confirmed the accumulation of lipids in adipocytes (Figure 7I) and the presence of apatitic calcium phosphate (Figure 7J) in the co-differentiation medium.

### Relative localization of adipocytes and mineralized matrix

**Figure 8:**
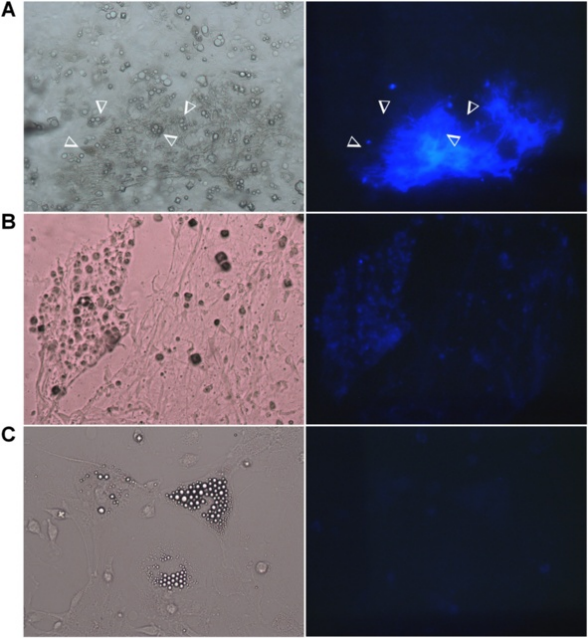
**Relative localization of adipocytes and mineralized matrix.** BMSCs were cultured for 14 days in co-differentiation **(A)**, osteogenic **(B)** or adipogenic **(C)** medium and treated with 30 μM of calcein blue. Cells were observed under white light (left) and under UV light (right). In left and right panels of **(A)**, the position of some adipocytes was indicated by the arrow heads. Fluorescence refers to mineralized matrix.

Cultures stained by calcein blue were observed under white light (left panel) and UV light (right panel) to localize adipocyte cells and mineralized matrix respectively (Figure 8). In co-differentiation medium, adipocytes were often observed very close to mineralized matrix (Figure 8A). Calcein blue staining of mineralization appeared less intense with usual osteogenic medium (Figure 8B). This confirmed results from calcium/protein assays and real time PCR experiments with osteoblastic markers. The very low fluorescent signal observed on cells differentiated in adipogenic medium confirmed the specificity of the fluorescence.

## Discussion

Mouse mesenchymal stem cell osteogenesis and adipogenesis are widely studied separately. The mechanisms and factors driving cells preferentially to one of these differentiation pathways are still discussed or to be identified. Because these two phenomena co-exist in the bone marrow, we attempted to reproduce both of them in a single culture condition, for further studies on their interactions.

Dex was chosen as a key factor because of its effects on osteoblastic and adipocytic differentiation capabilities [12-23,26,27]. Interestingly, Dex effects were shown to be dependent on cell differentiation stage. Indeed, in cells committed to the osteogenic lineage, such as MC3T3-E1 and ROS 17/2.8 cells, Dex stimulated osteogenesis [28,29]. The high time dependency of Dex effects on mineralization of human preosteoblast (SV-HFO) cultures was also demonstrated [27]. Conversely, in non-committed cells, such as C26 cells (clonal rat mesenchymal progenitor cell line), Dex had a stimulatory action on adipogenesis [30]. In addition, it was also demonstrated that Dex effects were widely dependent on its concentration. Particularly, the number of adipocytic cells derived from mouse BMSCs was higher with 10^−6^ M of Dex than with 10^−9^ M [31]. Thus, to create an environment in which both osteogenesis and adipogenesis occur, we induced osteogenesis by using a standard osteogenic medium, in which different doses of Dex were added to also induce adipogenesis.

BMSC population was chosen because it contains non-differentiated cells and cells partly committed to osteoblast or adipocyte lineages, as shown by the low levels of late differentiation markers of adipocyte (leptin) and osteoblast (osteocalcin) in cells cultured in growth medium. Our results demonstrated that Dex at 100 nM induced adipogenesis despite the presence of standard osteogenic factors. The emergence of adipocyte-like cells containing lipid droplets was accompanied by a rise in mRNA level of early as well as late adipocyte markers (PPARgamma, glut4, adiponectin and leptin). The true adipocyte commitment of these cells was supported by the absence of enhancement of adipocyte markers when Dex was added in the osteogenic medium for the last 2 days only.

Interestingly, Dex addition in osteogenic medium for 14 days also strongly increased the mineralization and the mRNA level of osteoblast markers (runx2, osterix and osteocalcin) when compared to cells differentiated in osteogenic medium without Dex. We checked that levels of mRNA cyclins (D1, E1 and B1) were not modified by Dex addition (Figure 6A). To strengthen the hypothesis that in our model Dex does not act through effects on cell proliferation, we performed western blot analysis of cyclin D1 which regulates the transition from G0 to G1 phases of cell cycle. Indeed, the results of these experiments suggested that there is no difference in cell proliferation level independently of medium and culture duration. This could be related to the cell culture confluence required in this protocol before inducing cell differentiation. Moreover, as for the adipocyte markers, we verified that this increase was not due to the enhancement of some osteoblast gene promoters by Dex. Our experiments showed that Dex added from day 12 to day 14 of osteoblast differentiation did not modify osteoblast marker mRNA levels. These results suggest that the higher levels of osteoblast markers were due to a true increase in osteoblast activity by Dex. Even though, it could also be supposed that these genes were no more sensitive to Dex at late days of osteoblastic differentiation. Taken together, all these results show that in our study, Dex strongly supported both differentiation pathways in the same culture medium.

Despite recent works showing that Dex treatment enhanced the alkaline phosphatase (ALP) mRNA level or activity of C3H10T1/2 cells [32] and rat mesenchymal cells [33,34], we did not find any difference in ALP mRNA level between osteogenic medium with Dex and osteogenic medium without Dex. This result could be explained by the fact that ALP is already strongly expressed in the standard osteogenic medium and thus could not be further enhanced by Dex addition. Indeed, we determined ALP mRNA levels more than 5 folds higher in co-differentiation medium than in growth medium, while in very similar osteogenic experiments only a 2 folds increase was shown [35].

In order to characterize the mineralization nodules found in the co-differentiation medium and to confirm lipid accumulation in the adipocytes, we used an original and complementary approach, “the Raman microspectroscopy” because it is a non-destructive method. Recently, its efficiency to characterize mineral content and to study MSC differentiation was shown [24,25]. In our study, Raman spectra with bands characteristic of apatitic calcium phosphate and of lipid accumulation were obtained in osteogenic and adipogenic cultures, respectively, and in the co-differentiation medium cultures. These Raman data further support the notion that in co-differentiation medium both adipocytes and osteoblasts are formed.

Our experiments with staining of mineralized matrix showed that in co-differentiation medium, adipocytes were often observed at proximity of this matrix. This observation led us to suppose that adipocytes and osteoblasts were able to develop in a close proximity. However, further investigations will be needed to determine more precisely the relative localization of adipocytes and mineralized nodules in this medium. Moreover, it would be useful to study this topology after several culture durations to establish if adipocytes were differentiated after or before mineralization. To hypothesize on the factors allowing both enhancements in this model, it is to note that whole bone marrow adherent cells were used. These cell populations might include pre-osteoblastic and pre-adipocytic cells as demonstrated by Post et al. [11], and by expression of adipocyte and osteoblast marker in growth medium (Figure 2). Interestingly, it was previously shown that Dex increased the adipocytic differentiation in adipogenic medium and osteoblastic differentiation in osteogenic medium [21], and that Dex can enhance expression of osteocalcin and aP2 (the fatty acid binding protein) in cultured rat BMSCs [36]. Taken together, these data lead to suppose that Dex potentiates the differentiation of cells already committed to a differentiation pathway.

Numerous *in vitro* studies described the balance between adipogenesis and osteogenesis of human, rat or mouse cells. Some of these studies showed that adipogenic factor addition in the osteogenic medium induced a decrease in osteoblast markers or functions. Other studies demonstrated that osteogenic factor addition induced a decrease in adipocyte markers or functions. From these data, authors concluded to a probable direct competition between these two differentiation pathways. Moreover, most of these studies were not done on the whole adherent cell population of bone marrow, but on mesenchymal progenitor or pluripotential cell lines. Then, another explanation could be that in a selected cell population a strong induction of one pathway compromises the initial one. In the present study, adipocyte lineage does not seem to increase at the expense of the osteoblast lineage, it can be hypothesized that adipocytes are derived from cells that would not be driven to the osteoblast lineage, even in pure osteogenic medium. Therefore, in the bone marrow these cells could be mesenchymal cells more or less committed in a non-osteoblastic differentiation pathway. Further investigations are needed to confirm this hypothesis and to characterize the underlying mechanisms. Despite we have obtained the differentiation of adipocytes and osteoblasts, our model represents some limitations because BMSC cultures do not reproduce the whole niche of bone marrow stroma, niche that remains to be determined.

Finally, the highest values of parameters of osteoblast and adipocyte differentiation were obtained for the same concentration of Dex. This suggests a positive link between the two phenomena in our model. It was previously described that Dex enhances leptin release from human adipose tissues [20,37,38] and that leptin has a direct pro-osteogenic effect [20,39]. Our study showed that leptin expression stayed at the growth medium level when cells were cultured in adipogenic medium with 100 or 500 nM of Dex, but it increased in the co-differentiation medium (Figure 2). First, this led us to suppose the involvement of the direct pro-osteogenic effect of leptin released from adipocytes, added to direct effects of Dex. Second, these results suggest that adipocytes induced in co-differentiation medium could be different from those induced in adipogenic medium but further investigations will be needed to characterize in greater detail these adipocytes.

## Conclusion

In summary this study demonstrated that the classical osteogenic medium added with 100 nM of Dex supports adipocyte and osteoblast differentiation of mouse BMSCs. The presence of both cell types was confirmed by several approaches. It was also shown that both differentiation lineages are enhanced in this medium when compared to classical adipogenic or osteogenic culture medium.

Moreover, this co-differentiation medium offers the opportunity to study BMSC differentiation capacities in an environment allowing both lineages and to study their interaction in great detail. This medium could also allow identifying new modulators of the osteoblast/adipocytes balance.

## Methods

### Primary bone marrow cell cultures

Female C57BL6 mice, 7 weeks of age, were purchased from Charles River (St Germain sur l’Abresle, France) and were acclimatized for 1 week under standard laboratory conditions (temperature 23 ± 1°C, humidity 50 ± 5%, water and standard food ad libitum). Immediately after sacrifice, femurs and tibias were removed and cleaned of connective tissue, the ends were cut, and the marrow was flushed with growth medium containing alpha Minimum Essential Medium (α-MEM) (PAN Biotech, Dutsher, Brumath, France) supplemented with 15% fetal calf serum (FCS) (PAN Biotech), 2 mM of glutamine (PAN Biotech), 50 IU/ml of penicillin (PAN Biotech), and 50 μg/ml of streptomycin (PAN Biotech). Single-cell suspensions were prepared in growth medium by passing the cells several times through graded needles. Cell density was determined using a Malassez counting chamber. For all experiments, cells were plated at a density of 2 × 10^6^ cells/cm^2^ and incubated at 37°C in 5% CO_2_. After 24 hours, non-adherent cells were removed and the medium was changed every 3 days until cell confluence. Mouse care and treatment were conducted in accordance with institutional guidelines in compliance with national law and policy. This study was approved by the Committee on the Ethics of Animal Experiments of Nord – Pas de Calais, France (Permit number: CEEA #022012).

### Osteogenic, adipogenic and co-differentiation media

Primary bone marrow-derived cells were induced to osteogenesis in the standard osteogenic medium composed by α-MEM 10% FCS supplemented with 50 μg/ml ascorbic acid (Sigma-Aldrich Corporation, St Quentin Fallavier, France) and 10 mM β-glycerophosphate (Sigma-Aldrich corporation) for up to 14 days and the medium was changed every 2 or 3 days.

Adipogenesis was induced in the standard adipogenic medium containing Dulbecco’s Modified Eagle Medium (DMEM) (PAN Biotech) 10% FCS supplemented with 10 μg/ml insulin/0,1 or 0,5 μM dexamethasone (depending on the experiment)/100 μM indomethacin/500 μM 3-isobutyl-1-methylxanthine (Sigma-Aldrich Corporation) for 4 days and then maintained in 10 μg/ml insulin/0,1 or 0,5 μM dexamethasone/5 μM pioglitazone (Sigma-Aldrich Corporation) for 10 days and the medium was changed every 2 or 3 days.

In order to obtain both adipocytes and osteoblasts in the same differentiation medium, cells were cultured in the standard osteogenic medium supplemented with different concentrations of Dex ranging from 50 nM to 150 nM. For these experiments, co-differentiation medium referred to osteogenic medium added with 100 nM of Dex.

### Oil red O staining and relative quantification

Cells were fixed in 2% paraformaldehyde for 15 min, washed with water, incubated with 60% isopropanol for 5 min and stained with newly filtered oil red O solution for 10 min at room temperature. To quantify staining, oil red O was extracted from the cells with isopropanol and absorbance of the solution was measured at a wavelength of 500 nm to determine the relative amount of dye.

### Mineralization staining

Mineralization staining was studied by two tests: alizarin red and calcein blue. For alizarin red, media were discarded and cells were fixed with 4% paraformaldehyde and stained with 2% alizarin red at pH 4.2. After 15 min of incubation at 37°C, cells were thoroughly washed with water.

For calcein blue (Sigma-Aldrich corporation), differentiating cells were treated with 30 μM of solution and the medium was changed every 3 days. Prior to microscope examination, cultures were washed with fresh medium and were fixed with 4% paraformaldehyde. Fluorescence was observed by using DAPI filter.

### Quantification of mineralization

Cells cultured for 14 days were harvested in a solution of PBS 1X (Phosphate Buffered Saline, PAN Biotech)/Triton 0.2%/HCl 6 M and disrupted by sonication. 5 μl of HCl 6 M was added to 95 μl of each sample and incubated overnight at 4°C. After centrifugation at 1500 × g for 5 min, the protein content was determined using the DC protein assay kit BioRad. The mineralization content was quantified as previously described in the work of Bruedigam et al. [40]. The results are shown as mM Ca^2+^/mg protein.

### Western blot analysis

Western blot analysis was performed as described previously [41]. For this study, rabbit polyclonal antibody (Cyclin D1) and mouse monoclonal antibody (ß-Actin) were used (Santa Cruz Biotechnology, Santa Cruz, CA, USA).

### RNA extraction

Total RNA was extracted from cultures with Extract-all (Eurobio, Les Ulis, France) according to the manufacturer’s protocol. Total RNA was quantified using the NanoDrop 2000 spectrophotometer (Thermo Scientific, Labteck, Palaiseau, France), and the integrity of RNA was controlled by the 28S:18S mRNA ratio after agarose gel electrophoresis. Contaminating DNA was removed from RNA samples by a 30 minute digestion at 37°C with DNase I (Roche Applied Science, Meylan, France).

### Reverse transcription and Real-time PCR

Reverse transcription and real-time PCR were performed as described previously in the work of Ghali et al. [41]. The sequences of the primers (TibMolBiol, Berlin, Germany) used for each of the analyzed genes are shown in the Table 2.

**Table 2:** **Primers sequences and conditions of PCR**

**Gene**	**Primer sequences**	**Annealing temperature**	**Product length**	**Genbank**
Runx2	F: GCCGGGAATGATGAGAACTA	62°C	200 bp	NM_001146038.2
	R: GGACCGTCCACTGTCACTTT			
OC	F: AAGCAGGAGGGCAATAAGGT	60°C	364 bp	NM_007541.3
	R: CGTTTGTAGGCGGTCTTCA			
OSX	F: CTAGTTCCTATGCTCCGACC	54°C	237 bp	NM_130458.1
	R: TCATCACATCATCATCGTG			
ALP	F:CAAAGGCTTCTTCTTGCTGGT	60°C	257 bp	NM_007431.3
	R: AAGGGCTTCTTGTCCGTGTC			
PPAR gamma −2	F: GGTGAAACTCTGGGAGATTCT	55°C	268 bp	NM_011146.3
	R: CAACCATTGGGTCAGCTCTTG			
Leptin	F: CTCATGCCAGCACTCAAAAA	62°C	197 bp	NM_008493.3
	R: AGCACCACAAAACCTGATCC			
Glut4	F: ACTCTTGCCACACAGGCTCT	62°C	174 bp	NM_009204.2
	R: AATGGAGACTGATGCGCTCT			
Adiponectin	F: CCCAGTCATGCCGAAGA	62°C	354 bp	NM_009605.4
	R: TACATTGGGAACAGTGACGC			
GAPDH	F: GGCATTGCTCTCAATGACAA	62°C	200 bp	NM_008084.1
	R: TGTGAGGGAGATGCTCAGTG			
18S	F: ATTCCGATAACGAACGAGAC	60°C	297 bp	NR_003278.3
	R: GCTTATGACCCGCACTTACT			
Cyclin D1	F: CGCACTTTCTTTCCAGAGTCA	55°C	74 bp	NM_007631.2
	R: AAGGGCTTCAATCTGTTCCTG			
Cyclin E1	F: TCAACGACACGGGTGAG	55°C	196 bp	NM_007633.2
	R: ATGTCGCACCACTGATAACCT			
Cyclin B1	F: CGCTCAGGGTCACTAGG	54°C	159 bp	NM_172301.3
	R: CTTCGCTGACTTTATTACCAA			

### Raman microspectroscopy

Cells cultured in growth, adipogenic, osteogenic or co-differentiation media were fixed in 4% paraformaldehyde. After rinsing, the fixed plates were analyzed with Raman microspectrometer LabRAM HR800 (Jobin-Yvon, France). The instrument was equipped with a XYZ motorized stage and a diode laser at 785 nm. The Raman spectrometer was coupled with microscope (BX40, Olympus).

Raman acquisitions were done with an immersion objective (Olympus LUMPLFL 100XW, numerical aperture = 1, Japan). The water immersion objective focused the laser on the cell within the micrometer scale. Spectral acquisition was made in the 300–1700 cm^−1^ range and spectral resolution was 4 cm^−1^. The Raman signal was collected by a multichannel CCD detector (1024 × 256 pixels). The acquisition time was set at 60s per spectrum. Raman spectra were processed using Labspec software (HORIBA, Jobin-Yvon, France). A Savitzky-Golay smoothing filter and polynomial baseline correction were applied to all Raman spectra.

Reference Raman spectra of adipocytes and mineralized nodules represented a 30 spectra of cells cultured in adipogenic medium and 30 spectra of cells cultured in osteogenic medium, respectively. To confirm the adipogenic and osteogenic phenotypes, spectra were compared with those described by Crane et al. [25] and Downes et al. [24].

The Raman spectra of undifferentiated cells (cells cultured in growth medium) and cell support (dish without cells) were also determined as negative controls.

To identify the adipocyte phenotype in the co-differentiation medium, adipocytes were selected according to the presence of lipid droplets. On these cells, 30 spectra were acquired and compared to reference Raman spectra of adipocytes. The presence of mineralized nodules was confirmed in this medium by acquiring 30 spectra on supposed mineralized area. These spectra were compared to reference Raman spectra from standard osteogenic medium. The same protocol was applied for cells without lipid droplets and without mineralized nodules. Obtained spectra were close to those from cells cultured in growth medium (undifferentiated cells). The Raman bands observed in the osteogenic, the adipogenic and the co-differentiation media and their assignments are summarized in the Table 1.

### Statistical analysis

All experiments were done in triplicate and repeated four times (with the exception of Raman analyses that were repeated three times). Mean values and S.E.M. (Standard Errors of the Mean) were calculated on the four independent experiments. The Mann-Whitney’s test for the statistical significance was performed by using GraphPad Prism software. Differences with p <0.05 were considered statistically significant.

## Abbreviations

MSC: Mesenchymal stem cells
BMSCs: Bone marrow stromal cells
Dex: Dexamethasone
Glut4: Glucose transporter type 4
AdipoQ: Adiponectin
O100: Osteogenic medium added with 100 nM of dexamethasone
Q-RT PCR: Quantitative reverse transcription polymerase chain reaction
OC: Osteocalcin
OSX: Osterix, ALP, Alkaline phosphatase
α MEM: Alpha minimum essential medium
FCS: Foetal calf serum
DMEM: Dulbecco’s modified eagle medium
DAPI: 4′-6-Diamidino-2-phenylindole
PBS: Phosphate buffered saline
HCl: Hydrochloride acid
Ca2^+^: Calcium
